# MSC Senescence-Related Genes Are Associated with Myeloma Prognosis and Lipid Metabolism-Mediated Resistance to Proteasome Inhibitors

**DOI:** 10.1155/2022/4705654

**Published:** 2022-11-23

**Authors:** Yang-Jia Cao, Yan-Hua Zheng, Qing Li, Jin Zheng, Li-Tian Ma, Can-Jun Zhao, Tian Li

**Affiliations:** ^1^State Key Laboratory of Experimental Hematology, National Clinical Research Center for Blood Diseases, Haihe Laboratory of Cell Ecosystem, Institute of Hematology & Blood Diseases Hospital, Chinese Academy of Medical Sciences & Peking Union Medical College, Tianjin 300020, China; ^2^Department of Hematology, The First Affiliated Hospital of Xi'an Jiaotong University, Xi'an, Shaanxi, China; ^3^Department of Hematology, Tangdu Hospital, Fourth Military Medical University (Air Force Medical University), Xi'an, Shaanxi, China; ^4^Department of Traditional Chinese Medicine, Tangdu Hospital, Fourth Military Medical University (Air Force Medical University), Xi'an, China; ^5^Department of Gastroenterology, Tangdu Hospital, Fourth Military Medical University (Air Force Medical University), Xi'an, China; ^6^School of Basic Medicine, Fourth Military Medical University (Air Force Medical University), 169 Changle West Road, Xi'an, China

## Abstract

**Background:**

Complex carcinogenic mechanisms and the existence of tumour heterogeneity in multiple myeloma (MM) prevent the most commonly used staging system from effectively interpreting the prognosis of patients. Since the microenvironment plays an important role in driving tumour development and MM occurs most often in middle-aged and elderly patients, we hypothesize that ageing of bone marrow mesenchymal stem cells (BM-MSCs) may be associated with the progression of MM.

**Methods:**

In this study, we collected the transcriptome data on MM from The Cancer Genome Atlas (TCGA) and the Gene Expression Omnibus (GEO) databases. Differentially expressed genes in both senescent MSCs and MM tumour cells were considered relevant damaged genes. GO and KEGG analyses were applied for functional evaluation. A PPI network was constructed to identify hub genes. Subsequently, we studied the damaged genes that affected the prognosis of MM. Least absolute shrinkage and selection operator (lasso) regression was used to identify the most important features, and a risk model was created. The reliability of the risk model was evaluated with the other 3 GEO validation cohorts. In addition, ROC analysis was used to evaluate the novel risk model. An analysis of immune checkpoint-related genes, tumour immune dysfunction and exclusion (TIDE), and immunophenotypic scoring (IPS) were performed to assess the immune status of risk groups. pRRophetic was utilized to predict the sensitivity to administration of chemotherapeutic agents.

**Results:**

We identified that MAPK, PI3K, and p53 signalling pathways were activated in both senescent MSCs and tumour cells, and we also located hub genes. In addition, we constructed a 14-gene prognostic risk model, which was analysed with the ROC and validated in different datasets. Further analysis revealed significant differences in predicted risk values across the International Staging System (ISS) stage, sex, and 1q21 copy number. A high-risk group with higher immunogenicity was predicted to have low proteasome inhibitor sensitivity and respond poorly to immunotherapy. Lipid metabolism pathways were found to be significantly different between high-risk and low-risk groups. A nomogram was created by combining clinical data, and the optimization model was further improved. Finally, real-time qPCR was used to validate two bortezomib-resistant myeloma cell lines, and the test confirmed that 10 genes were detected to be expressed in resistant cell lines with the same trend as in the high-risk cohort compared to nonresistant cells.

**Conclusion:**

Fourteen genes related to ageing in BM-MSCs were associated with the prognosis of MM, and by combining this genotypic information with clinical factors, a promising clinical prognostic model was established.

## 1. Introduction

Multiple myeloma (MM) is a cancer entailing heterogeneous clonal proliferation of plasma cells and accounts for more than 10% of all haematologic malignancies [1]. The main clinical presentation of MM is the development of osteolytic bone lesions, hypercalcaemia, renal insufficiency, and manifestations associated with bone marrow failure [2]. Although the continuous emergence of new treatments has led to higher survival rates for patients with MM, it remains incurable for several reasons, including relapse or refractory disease, drug resistance, and disease-related organ dysfunction. Due to the high heterogeneity of tumour cells, the staging and classification of multiple myeloma have been keys to individualized and precise treatment. Currently, prognostic assessment of patients with multiple myeloma relies on tumour load markers and cytogenetics. However, in clinical practice, we often find that neither the International Staging System (ISS) nor the Durie–Salmon (DS) staging accurately interpret the prognoses of patients. Especially for elderly patients, cytogenetics contributes progressively less to risk, and tumour cell load is not a good measure of prognosis [3].

Smouldering multiple myeloma (SMM) is very likely to progress to active multiple myeloma (with a probability of approximately 73%) [4]. Mice with a smouldering phenotype have been found to produce higher levels of serum immunoglobulin and exhibit decreased bone density only later in life [5], suggesting that ageing may accelerate disease development. A recent study found that undetermined significance (MGUS) and SMM development into MM are not only dependent on intrinsic PC characteristics but also influenced by biology of the surrounding microenvironment [6, 7]. Further research on the tumour microenvironment will be necessary to unravel mechanisms underlying myeloma initiation and development.

Mesenchymal stem cells (MSCs) play an important role in the bone marrow microenvironment. Bone marrow mesenchymal stem cells (BM-MSCs) are adult stromal cells of mesodermal origin with the ability to modulate immune system responses, self-renewal, injury repair, and multipotent differentiation [8]. However, low cell proliferation capacity and a decline in differentiation potential appear in MSCs with donor ageing [9]. The weakening of MSCs leads to poor osteogenic differentiation and disordered immunoregulation [7]. Malignant MM cells cooperate with stromal cells to secrete cytokines and growth factors that are responsible for the biological and clinical manifestations of the disease. Even after successful antitumour therapy, the inflammatory state of the bone marrow persists [10]. Tumour treatment remains limited to killing tumour cells, but this may also cause damage to microenvironmental cells. Therefore, it is important to consider the interaction between tumour cells and other cells in the microenvironment. Effective therapeutic interventions must target both myeloma cells and the BM niche.

BM-MSCs could be more than victims; they may constitute the cause of microenvironmental inflammation in the development of myeloma [11]. The inflammatory microenvironment promotes multiple myeloma cell growth and resistance to conventional therapies [12]. To date, few studies have demonstrated a link between MSC ageing and MM progression. In addition, the significance of microenvironment ageing-related genes in MM prognosis has not been determined. Here, we explored the significance of MSC-based ageing characteristics in MM patients by examining the expression of MSC ageing-related genes in MM from a comprehensive gene expression database (GEO) and related clinical information using bioinformatics analysis. The predictive value of the model was further validated using external GEO sequences.

## 2. Materials and Methods

### 2.1. Source and Description of the Gene Expression Dataset

**Figure 1:**
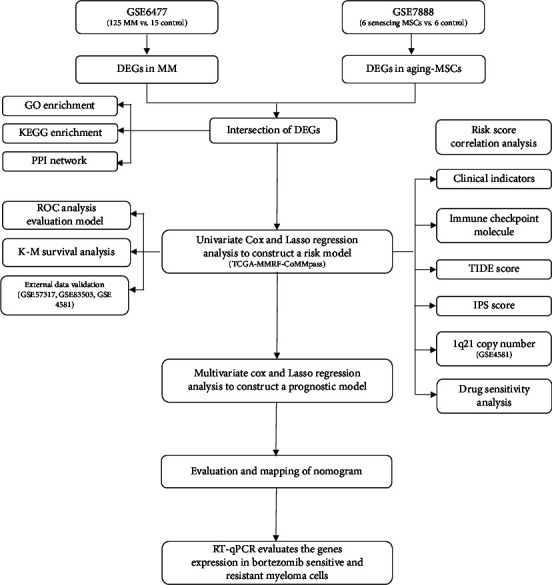
The flowchart of this study.

Transcript levels and matched clinical information were collected from the GEO (https://www.ncbi.nlm.nih.gov/geo/) database and The Cancer Genome Atlas (TCGA) database (https://portal.gdc.cancer.gov/). GEO accession number GSE7888 contains cultivated BM-MSCs samples from 6 healthy humans and 6 matched senescent stage samples obtained from an in vitro culture. Multiple myeloma expression profiles were obtained from accession number GSE6447, with 125 MM (excluding MUGS) and 15 healthy donor plasma cell samples. The MSC senescence-related gene prognostic signature was reconstructed based on the training dataset (MMRF-CoMMpass downloaded from TCGA) (*n* = 858). The other MM data were obtained from GEO accession numbers GSE57317 (*n* = 55), GSE83503 (*n* = 586, 16 patients with missed visits were excluded), and GSE4581 (*n* = 414) to validate the outcome. A flowchart of the investigation is depicted in Figure 1.

### 2.2. Acquisition of Mesenchymal Stem Cell Senescence-Related Genes and Differentially Expressed Genes (DEGs) in Normal Plasma Cells and Multiple Myeloma

The relative gene expression of MSC senescence-related genes was normalized and identified using the “limma” package with adj. *p* value <0.05 as the screening condition. Genes differentially expressed between MM samples and normal samples were identified by using the “limma” *R* package. Differentially expressed genes (DEGs) were identified using adj. *p* value <0.05 as the screening condition.

### 2.3. Enrichment Analysis and Protein Interaction Network Construction

The intersection of genes differentially expressed between MM and normal plasma cells with MSC senescence-related genes was examined. The roles of intersecting genes in biological pathways were explored using the “clusterProfiler” package. Intersected DEGs were entered into the STRING database to obtain a protein-protein interaction (PPI) network, which was visualized using Cytoscape software. Differential gene enrichment analysis for high and low-risk groups was obtained from the Metascape database.

### 2.4. Construction of the Risk Model

The samples in TCGA-MMRF-COMMPASS were used as the training set, and genes significantly associated with survival were identified using univariate Cox proportional hazards analysis. The candidate genes obtained from one-way Cox regression analysis were subjected to lasso regression analysis using the R package “glmnet.” The risk model was constructed based on the expression of genes obtained from lasso regression. Risk scores were calculated, and patients were classified into high-risk and low-risk groups according to the median risk score. Differences in survival between patients in high-risk and low-risk groups were analysed using Kaplan–Meier (KM) curves. ROC curve analysis was used to evaluate the performance of risk scores for predicting patient survival status.

### 2.5. Evaluation of Risk Model Prediction Performance

The constructed model was validated using the validation set (GEO accession numbers GSE57317, GSE83503, and GSE4581), and based on the model, the risk score was calculated for each patient. According to the median risk score, patients were divided into a high-risk group and a low-risk group, and survival differences between patients in the high-risk and low-risk groups were analysed using Kaplan–Meier (KM) curves. ROC curve analysis was also used to evaluate the performance of risk scores in the prediction of patient survival status.

### 2.6. Evaluation of the Sensitivity of Chemotherapeutic Agents

The 50% inhibition concentration (IC50) values of 138 drugs were inferred using the “pRRophetic” algorithm, comparing the IC50 of chemotherapeutic agents in the high-risk and low-risk groups.

### 2.7. Immune-Related Characteristics in the Low-Risk and High-Risk Score Groups

A total of 282 immune checkpoint-related genes (ICRGs) were collected based on a previous study. The differences in ICRGs between the high-risk and low-risk groups were analysed. Tumour immune dysfunction and exclusion (TIDE) was used to evaluate the prognostic effect of immune checkpoint inhibition therapy. Analytical data were based on TPM data of all tumour samples (*n* = 858) from the TCGA dataset after log_2_ (TPM+1) transformation. The data were normalized using the mean of all samples as a control, as required by official documents. Processed data were uploaded to the TIDE website (https://tide.dfci.harvard.edu). TIDE analysis results were obtained. An analysis of correlations between TIDE and risk scores was conducted, and the differences in TIDE scores between the high-risk and low-risk groups were examined. Moreover, immunophenotypic scoring (IPS) of all 858 tumour samples was performed using the “IOBR” package.

### 2.8. Correlations with Clinical Indicators and Independent Prognostic Analysis

For the GSE4581 dataset, we compared risk scores between patients with a 1q21 copy number greater than 2 and patients with a normal copy number, and we used the TCGA dataset to compare risk scores by sex, race, age, and ISS stage.

### 2.9. Establishment of a Nomogram and Statistical Analysis

Using the “rms” package in R, we built clinical prognostic models based on the TCGA dataset using the risk scores generated in the previous phase and clinical data from patients, including sex, ethnicity, and stage. Prognostic models were constructed using univariate Cox analysis and multivariate Cox regression, respectively. The accuracy of the nomogram was predicted by plotting calibration curves over time. Multivariate Cox regression analysis was also performed to determine whether the prognostic risk score model could be employed as an independent predictor of OS in MM. The nomogram's prognostic value was then computed using AUC values from online ROC curves. All data analyses employed *R* software (version 4.1.3 for Windows, https://www.R-project.org). Differences between the two groups were evaluated using the Wilcoxon test. Kaplan–Meier analysis was applied to evaluate survival differences between the low-risk and high-risk score groups. A value of *p* < 0.05 was considered statistically significant.

### 2.10. Cell Lines and Cultures

In this experimental study, we used the RPMI-8226 and OMP-2 cell lines from Fourth Military Medical University, and RPMI-8266 bortezomib-resistant cells and OMP-2bortezomib-resistant cells were previously developed and cultured in the presence of bortezomib (Selleckchem PS-341) (Houston, TX, USA).

### 2.11. Quantitative Real-Time Polymerase Chain Reaction Assessment and Statistical Analysis

Total RNA was isolated from the bortezomib-resistant and nonresistant RPMI-8226 and OMP-2 according to the TRIzol manufacturer's protocol (Invitrogen; Thermo Fisher Scientific, Inc.). RNA was reverse transcribed to cDNA using Transcriptor First Strand cDNA Synthesis Kit (Roche Diagnostics) according to the manufacturer's protocols. Quantitative real-time polymerase chain reaction (qRT-PCR) was performed with SYBR Green PCR Master Mix (Roche Diagnostics).  Table1 shows the primer sequences used in this experiment. The qPCR program cycling parameters were: qPCR was performed as follows: initial denaturation for 30 sec at 95°C, followed by 40 cycles of 95°C for 15 sec and 60°C for 30 sec. GADPH was used as an internal control, and data analysis was performed by 2^−ΔΔCT^ to calculate the fold change for relative expression. Data were presented as a mean ± standard deviation. Statistical analysis was performed with the GraphPad Prism 8.0.3 software. The mean values of two groups were compared by Student's *t*-tests. *P* < 0.05 was considered statistically significant.

## 3. Results

### 3.1. Identification of the Intersection of Genes Differentially Expressed in Aged BM-MSCs and Multiple Myeloma

**Figure 2:**
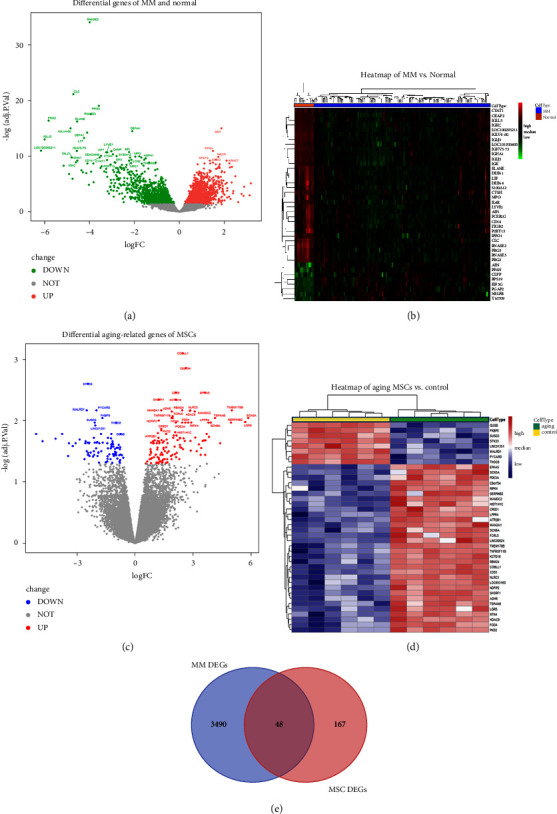
Identification of damage-associated genes. (a) A volcano map of genes differentially expressed in MM. Red dots indicate upregulated genes. Green dots indicate downregulated genes. Grey indicates nonsignificant differentially expressed genes. (b) A heatmap of differential gene expression between MM and normal control. Red indicates high expression, and green indicates low expression. (c) A volcano map of genes differentially expressed in MSC senescence. Red dots indicate upregulated genes. Blue dots indicate downregulated genes. Grey indicates nonsignificant differentially expressed genes. (d) A heatmap of differential gene expression in MSC senescence. Red indicates high expression, and blue indicates low expression. (e) A Venn diagram of MSC ageing genes and genes differentially expressed in MM.

Differentially expressed genes were identified in CD138+ plasma cells from multiple myeloma patients (*n* = 125) and plasma cells from healthy individuals (*n* = 15) in data from GEO accession number GSE6477. A total of 3568 differentially expressed genes (MM vs. control) were identified, of which 1842 were upregulated and 1726 were downregulated. The volcano map and the gene heatmap of MM DEGs are plotted in Figures 2(a) and 2(b).

There were 217 differentially expressed genes identified (MSCs in the senescing stage vs. MSCs in the early stage), with 128 genes upregulated and 89 genes downregulated. A volcano map and a heatmap of genes differentially expressed in ageing MSCs are shown in Figures 2(c) and 2(d).

The intersection of differentially expressed MM and MSC genes included a total of 48 genes. Venn diagram mapping is shown in Figure 2(e). Since senescence is defined by the buildup of damage [13], to simplify terminology, we label these damage-related genes.

### 3.2. Enrichment Analysis of Damage-Related Genes and Construction of the PPI Network

**Figure 3:**
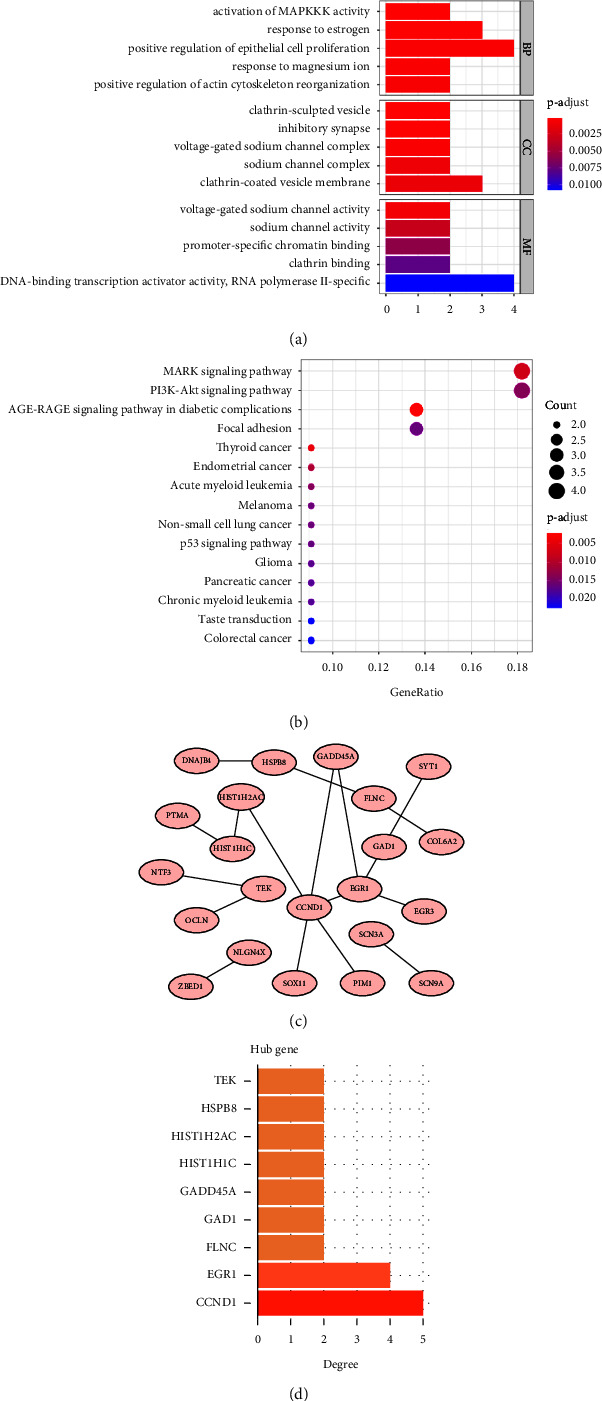
Functional enrichment of damage-associated genes and screening for hub genes. (a) GO enrichment of damage-associated genes. (b) KEGG enrichment of damage-associated genes. (c) PPI network diagram. (d) Hub genes displayed according to a degree.

A total of 48 damage-related genes obtained were subjected to GO enrichment. These genes were found to be enriched in biological processes (BPs), such as activation of MAPK activity, oestrogen response, positive regulation of epithelial cell proliferation, response to magnesium ions, and positive regulation of actin cytoskeleton reorganization. Furthermore, lattice protein vesicles, inhibitory synapses, voltage-gated sodium channel complexes, sodium channel complexes, lattice protein vesicle membranes, and other cellular components (CCs) were enriched. The main molecular functions (MFs) included voltage-gated sodium channel activation, sodium channel activity, catalytic-specific chromosome binding, and activation of RNA polymerase II-specificDNA-binding transcriptional activators (Figure 3(a)).

According to KEGG enrichment analyses, differentially expressed genes were mainly involved in the MAPK signalling pathway, PI3K-Akt signalling pathway, acute myeloid leukaemia, p53 signalling pathway, chronic myeloid leukaemia, and other signalling pathways (Figure 3(b)).

Damage-related genes were entered into the STRING database to obtain the protein-protein interaction (PPI) network (Figure 3(c)). Genes with a degree greater than 2 were used as hub genes. These hub genes were TEK, HSPB8, HIST1H2AC, HIST1H1C, GADD45A, GAD1, FLNC, EGR1, and CCND1 (Figure 3(d)).

### 3.3. Prognostic Risk Modelling of Multiple Myeloma

**Figure 4:**
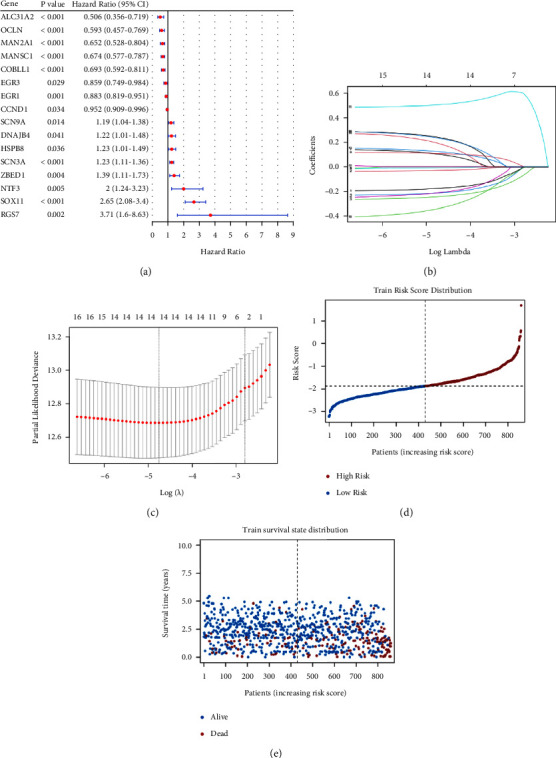
Construction of the risk model. (a) Univariate Cox regression analysis to screen for prognosis-related genes. (b) A curve of changes in the *λ*-value from lasso analysis. (c) The confidence interval for the value of *λ*. (d) Risk score distribution of the training set. (e) Survival state distribution of the training set.

A total of 858 samples with clinical information from TCGA-MMPF-COMMPASS were used as the training set, and a one-way Cox analysis was performed on 48 damage-related genes to screen out 16 genes associated with prognosis. The forest diagram is shown in Figure 4(a).

Lasso regression is a statistical method for obtaining a more refined model by constructing a penalty function, compressing some regression coefficients, reducing data dimensionality, and avoiding multicollinearity and overfitting in multiple regression models (Figures 4(b) and 4(c)). Sixteen candidate genes identified by one-way Cox regression analysis were subjected to lasso regression analysis. Fourteen genes whose regression coefficients were not penalized to 0 were finally obtained using lambda.min = 0.008623835 and 10-fold cross-validation: COBLL1, CCND1, MANSC1, MAN2A1, EGR1, SLC31A2, HSPB8, SCN3A, SCN9A, SOX11, RGS7, NTF3, OCLN, and ZBED1.

The risk model was constructed based on the expression of 14 genes obtained by lasso regression. The coefficient from the linear term of the lasso regression model was used to calculate the risk score Risk score = ∑_*i*=1_^*n*^*βi∗xi*, where *β*_*i*_ is the coefficient and *x*_i_ is the sample expression corresponding gene *i*. The coefficients for all 14 genes are calculated and shown in Table 2.

The formula was used to calculate risk scores for 858 patients, and patients were divided into the high-risk group (*n* = 429) and the low-risk group (*n* = 429) based on the median risk score. The distribution of patients' risk scores and survival status is shown in Figures 4(d) and 4(e).

### 3.4. Performance Evaluation of Risk Model Prediction

**Figure 5:**
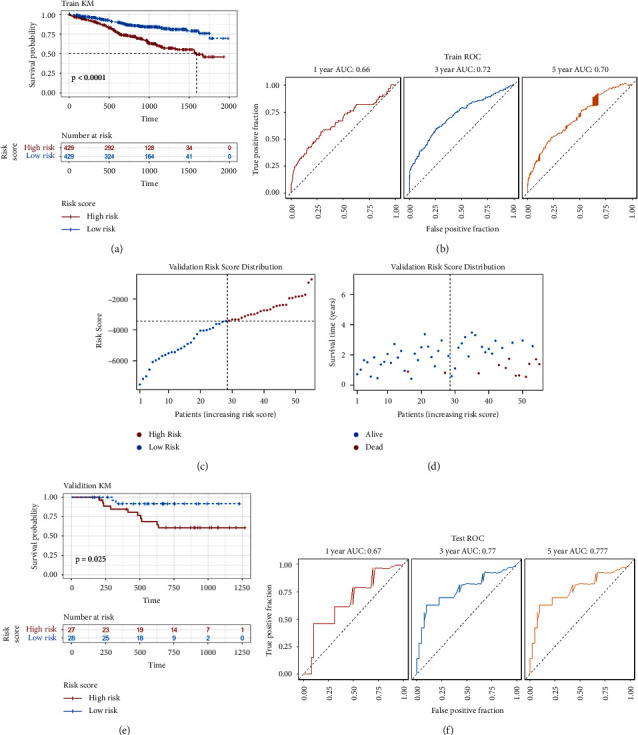
Evaluation of risk model prediction performance. (a) The training set Kaplan–Meier curve. (b) An ROC curve of the training set. (c) Risk score distribution of the validation set. (d) Survival state distribution of the validation set. (e) The validation set Kaplan–Meier curve. (f) An ROC curve of the training set.

The difference in survival between the high-risk and low-risk groups in the training set was analysed using the Kaplan–Meier (KM) curve. As shown in Figure 5(a), log-rank analysis revealed a significant difference in survival between the two groups (*p* < 0.05). Additional validation with two datasets. There was no median follow-up time in this dataset GSE83503, we removed the 16 samples of the missing visit data and used the chi-square test to calculate the survival rate in the high-risk and low-risk groups, and the results showed that the mortality rate was significantly higher in the high-risk group than in the low-risk group (*p* < 0.05). GSE4581 (*n* = 414) showed significant differences in survival between the high-risk and low-risk groups distinguished by the model constructed in this study (Figures S1A and S1B).

ROC curve analysis was also used to evaluate the performance of risk scores in the prediction of patient survival status, and ROC curves were plotted for 1, 3, and 5 years. The results are shown in Figure 5(b). The results showed that the AUC exceeded 0.66, indicating that the risk score could accurately predict the survival status of patients.

The constructed model was validated using the validation set (*n* = 55). Combined with the model, risk scores were calculated for each patient. Patients were divided into the high-risk group (*n* = 27) and the low-risk group (*n* = 28) based on the median risk score. The distribution of risk scores and survival status of patients are shown in Figures 5(c) and 5(d).

The Kaplan–Meier (KM) curve was used to analyse the difference in survival between the high-risk and low-risk groups of patients in the validation group. Log-rank analysis showed a significant difference in survival between the two groups (*p* < 0.05), as shown in Figure 5(e).

The performance of risk scores for predicting patient survival status was also evaluated using ROC curve analysis, and the 1-year, 3-year, and 5-year ROC curves were plotted, with the results shown in Figure 5(f). The AUC exceeded 0.67, indicating that the model was capable of accurate prediction.

### 3.5. Immune-Related Characteristics in the Low-Risk and High-Risk Score Groups

**Figure 6:**
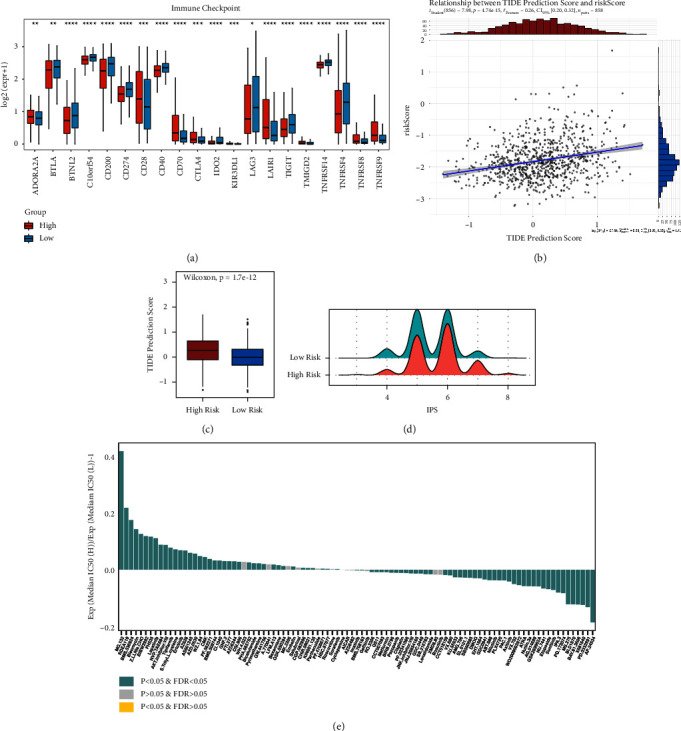
Immune-related characteristics and chemotherapy drug sensitivity in the TCGA dataset. (a) Differential immunoassay point molecules. (b) Correlation analysis of TIDE scores and risk scores. (c) A boxplot of differences in TIDE scores between the high-risk and low-risk groups. (d) A mountain plot of the difference in IPS scores between the high-risk and low-risk groups. (e) A histogram of the IC50 ratio for the high-risk and low-risk groups. The green column indicates a statistically significant difference.

The differences in immune checkpoint molecules between the high-risk and low-risk groups were then examined. The TCGA-MMRF-COMMPASS dataset corresponded to 46 genes related to immune checkpoint molecules. Using the Wilcoxon test (*p* < 0.05), 20 immune checkpoint molecules were found to differ significantly between the high-risk and low-risk groups: ADORA2L, BTLA, BTNL2, C10orf54, CD200, CD274, CD28, CD40, CD70, CTLA4, IDO2, KIR3DL1, LAG3, LAIR1, TIGIT, TMIGD2, TNFRSF14, TNFRSF4, TNFRSF8, and TNFRSF9 (Figure 6(a)). Markers representing T cell exhaustion, such as CD274 (PD1), LAG3, CTLA4, and TIGIT, were highly expressed in the low-risk group, which predicts that immunotherapy will not be effective for patients in the high-risk group.

A correlation analysis was conducted between the TIDE scores and risk scores of the TCGA-MMRF-COMMPASS dataset, and the differences in TIDE scores between the high-risk and low-risk groups were examined.  Figure 6(b) shows the distributions of TIDE and risk scores, both of which conform to a normal distribution. Therefore, Pearson's coefficient was used for correlation analysis. The correlation coefficient was 0.28 with a highly significant *p* value, which indicated that the risk score and TIDE obtained from the previous analysis were strongly correlated, and as the risk score increased, the TIDE score also increased, which implied poor efficacy of immune checkpoint blockade therapy (ICB) in the high-risk group and short survival after receiving ICB treatment. Furthermore, TIDE scores were significantly different between the high-risk and low-risk groups, as shown in Figure 6(c), with a higher TIDE score in the high-risk group. This is consistent with previous findings.

Immunophenotypic scoring (IPS) was performed on all 858 tumour samples. The results were plotted on a mountain plot (Figure 6(d)); overall, the IPS in the high-risk group was higher than that in the low-risk group, indicating high immunogenicity.

### 3.6. High-Risk Group Is Resistant to Proteasome Inhibitors and Associated with the Lipid Metabolism Pathway

**Figure 7:**
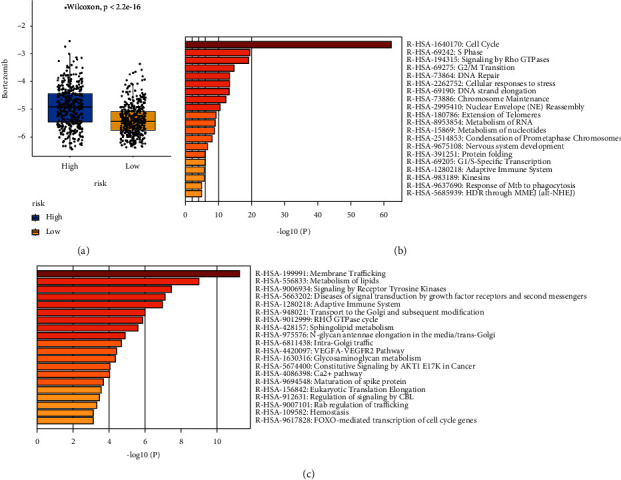
Differential bortezomib resistance and differential gene enrichment in the high and low-risk groups of GSE4581. (a) A boxplot of differences in predicted bortezomib IC50 between the high-risk and low-risk groups. (b) Enrichment pathways of genes upregulated in myeloma in the high-risk group than in the low-risk group. (c) Enrichment pathways of genes downregulated by myeloma in the high-risk group than in the low-risk group.

The half-maximal inhibitory concentration (IC50) was calculated to predict the treatment response to chemotherapy drugs in the cohort from TCGA. 137 chemotherapeutic agents were evaluated in the high-risk and low-risk groups of patients in the TCGA dataset, and MG.132 (a proteasome inhibitor) was found to be least sensitive to tumour cells in the high-risk group, while some CDK inhibitors and multitarget kinase inhibitors had lower drug sensitivity in the high-risk group (Figure 6(e)). Additionally, in the GSE4581 dataset, the low-risk group was more sensitive to bortezomib than the high-risk group (Figure 7(a)). We further analysed differential genes between the high-risk and low-risk groups and found that genes upregulated in the high-risk group were mainly enriched in cell cycle-related pathways compared to the low-risk group (Figure 7(b)) and that genes downregulated in the high-risk group were mainly enriched in membrane trafficking pathways and metabolism of lipids compared to the low-risk group (Figure 7(c)). This result suggests that the high-risk group, which was divided by our model, is resistant to proteasome inhibitors and associated with lipid metabolism pathways.

### 3.7. Risk Prediction Models Are Independent of Patient Clinical Characteristics

**Figure 8:**
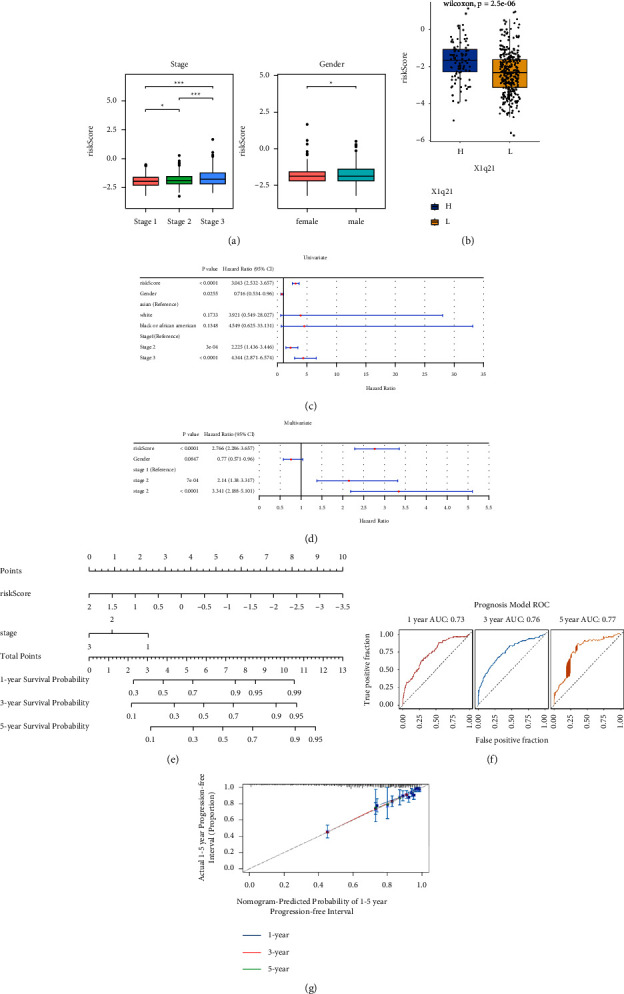
Clinical features and nomogram construction. (a) A boxplot of differences in risk scores across clinical indicators in the TCGA cohort. (b) A boxplot of differences in risk scores across the 1q21 copy number. Group H represents a 1q21 copy number greater than 2, and Group L represents a 1q21 copy number less than 2. (c) Univariate Cox analysis of prognostic clinical indicators. (d) Multivariate Cox analysis of prognostic clinical indicators. (e) Nomogram to predict survival in MM patients. (f) An ROC curve of the prognostic model. (g) The prognostic model evaluated using 1-year, 3-year, and 5-year calibration curves. The *x*-axis shows survival anticipated from the column plots, while the *y*-axis shows actual survival.

The risk score for the TCGA-MMRF-COMMPASS dataset was calculated, and the variation in risk scores with clinical indicators was analysed. The results showed that risk scores differed significantly among ISS stages and sexes (Figure 8(a)), which is consistent with clinical observations. In the GSE4581 dataset, the risk scores of patients with a 1q21 copy number greater than 2 were significantly higher than those of patients with a normal copy number (Figure 8(b)).

Univariate Cox analysis screened for clinical factors associated with prognosis, and the effect of race was not significant (Figure 8(c)). Among factors associated with OS in univariate analysis, including ISS stage, sex, race, and risk score, only the ISS stage, sex, and risk clinical score were independent predictors of OS in multivariate analysis (Figure 8(d)). Finally, the nomogram plot is shown in Figure 8(e). To evaluate the clinical prognostic model, ROC curve analysis (Figure 8(f)) and calibration curves (Figure 8(g)) suggested that the model was capable of accurate prediction.

### 3.8. Genes Expressed in Bortezomib-Resistant Cell Lines with the Same Trend as in the High-Risk Cohort Compared to Nonresistant Cells

**Figure 9:**
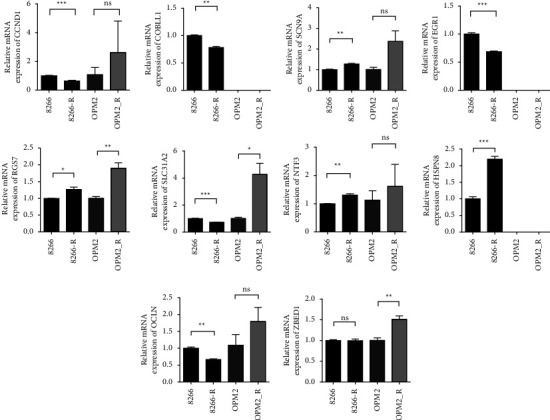
The RT-qPCR results. CCND1, COBLL1, EGR1, OCLN, and ZBED1 expression were low in bortezomib-resistant cell lines, and SCN9A, RGS7, NTF3, HSPB8 and ZBED1 expression were high in bortezomib-resistant cell lines (^*∗*^*p* < 0.05, ^*∗∗*^*p* < 0.01, ^*∗∗∗*^*p* < 0.001, ^*∗∗∗∗*^*p* < 0.0001, ns: no statistical significance, 8266-R: bortezomib-resistant RIPM-8266, OPM2_R: bortezomib-resistant OPM-2).

We evaluated mRNA levels of 14 genes in myeloma cell lines. In agreement with the gene model, compared with bortezomib-sensitive myeloma cell lines (negative control), there was an increase in mRNA expression of SCN9A, RGS7, NTF3, HSPB8, and ZBED1 and a decrease in CCND1, COBLL1, EGR1, OCLN, and ZBED1 mRNA expression of bortezomib-resistant cell lines (Figure 9). The expression trend of these genes was consistent with the high-risk group gene expression trend predicted by the model. SOX11 and SCN3A were not detected in these cell lines because their expression was too low.

## 4. Discussion

Multiple myeloma has a complicated pathogenesis, and mechanisms underlying its occurrence and progression remain largely unknown. For decades, resistance to drugs (particularly bortezomib), relapse, refractory disease, and inconsistency in the treatment of older individuals have been bottlenecks that hinder myeloma treatment. Furthermore, most of the genetic complexity of MM might be present at asymptomatic stages, but how initiating plasma cell clones acquire the potential for oncogenic transformation to MM is a crucial factor that has eluded researchers [14]. There is mounting evidence that multiple myeloma is linked to abnormal mRNA expression, which is connected to the occurrence, progression, and prognosis of the disease. Individual patient survival remains variable and cannot be accurately predicted using current prognostic models [15–18]. Current mRNA-based prognostic models are primarily concerned with identifying special tumour subtypes and exploring differential gene expression or activation pathways in tumour cells. Currently, the influence of the tumour microenvironment on tumour progression and drug resistance has become the focus of research. Treatment modalities that take the microenvironment into account may be more helpful for patient survival.

A growing number of studies confirm that the bone marrow microenvironment is not always passive in the development of tumours [19]. The inflammatory environment provides conditions for tumour induction and promotion, especially in myeloma, and MSCs play a crucial role as the niche of the microenvironment. According to Schinke et al., markers of MSCs are independent prognostic factors for MM and SMM, and microenvironmental immune dysfunction caused by MSCs plays a key role in disease progression [6]. Single-cell sequencing also confirmed that antitumour induction therapy fails to restore bone marrow inflammation, predicting a role for mesenchymal stromal cells in disease persistence [10]. Furthermore, impaired osteogenic differentiation of BM-MSCs is an important cause of myeloma bone disease [20]. Bone disease is one of the most prominent clinical symptoms of MM patients, affecting 80% of MM patients, and seriously affects the quality of life and survival time of patients [21]. However, a few drugs are currently available to target bone regeneration and treat adult bone weakness; pathway-based tumour treatment options may not be sufficient to improve bone disease. For example, inhibition of the Wnt signalling pathway blocks tumour progression while obstructing osteogenesis [22]. To better improve patient survival, therapy should not be limited to targeting tumour cells only. Bidirectional interferences between microenvironmental cells and the immune system may be potential targets for anticancer drugs. Microenvironmentalcell-targeted therapy as a possible systemic anticancer effect is receiving increasing attention [23]. MSCs play a very important immunomodulatory role in the tumour microenvironment, but the immunomodulatory capacity of MSCs depends on the type and intensity of inflammatory signals they receive. A high inflammatory state causes MSCs to produce T cell suppression, while a low inflammatory state causes MSCs to produce T cell activation [24]. Ageing MSCs secrete more inflammation-associated cytokines, and inflammatory cues may scramble the delicate balance of regulatory networks necessary to govern tissue-specific regeneration and remodelling [25]. Since multiple myeloma primarily affects elderly individuals, we speculate that ageing MSCs play an important role in the tumour microenvironment. We designed this study to identify genes coexpressed by tumour cells and senescent BM-MSCs in an attempt to provide evidence for a link between MSC senescence and MM progression. This research may shed light on future research for the treatment of MM. Perhaps by targeting these genes, it is possible to eliminate tumour cells while also improving the function of BM-MSCs.

In this study, we discovered a set of gene signatures that are essential in predicting MM progression and linked to MSC senescence, suggesting that early prevention of ageing in MSCs could help slow disease progression. The MAPK pathway and DNA repair pathway-related genes have been shown to be independent prognostic factors for high-risk SMM [26], and these pathways have been previously shown to be involved in the progression of myeloma [27–29]. The genes that we screened were also mainly the enriched MAPK pathway, PI3K pathway, and p53 signalling pathway, which further illustrates the relevance of our results to the progression and prognosis of myeloma. Further analysis revealed poor efficacy of immune checkpoint blockade therapy (ICB) in high-risk patients with strong immunogenicity, which suggests that in high-risk patients, the bone marrow microenvironment is extremely inflammatory and resistant to immune checkpoint drugs. We also found that patients with a copy number of 1q21 larger than 2 had a higher risk score, which confirms that grouping based on our model is reasonable. Chemotherapy drug sensitivity analysis shows that high-risk patients are less responsive to proteasome inhibitors. Numerous studies have confirmed the close relationship between MM drug resistance and MSCs. Tumour drug resistance is affected by MSCs through adhesion and paracrine effects [30]. Interestingly, in this study, myeloma cells from patients in the high-risk group were found to express fewer lipid metabolism-related genes than those in the low-risk group. Recent reports confirmed that abnormal lipid accumulation in multiple myeloma cells was enhanced by proteasome inhibitors; lipid-lowering drugs and MG-132 exerted a synergistic effect to kill multiple myeloma cells [31, 32]. These findings suggest that the combination of a lipid metabolism target with a broad-spectrum proteasome inhibitor may be effective in the presence of proteasome inhibitor resistance in high-risk patients as predicted in our model. Finally, we verified 14 genes in the model by RT-qPCR and found that the expression trend of 10 genes in bortezomib-resistant myeloma cell lines was consistent with our prediction. This suggests that these high-risk tumours are associated with drug resistance and that there is an urgent need to develop new drugs or new drug combinations to treat these high-risk patients. In the analysis of gene function, SCN9A, NTF3, and RGS7 tended to promote cell proliferation and migration. More experiments are needed in the future to further explore the role of these genes in MM. Perhaps therapies that target these genes will help improve tumour resistance and patient survival. What is noteworthy is that although SOX11 was not detected, it has been shown to be an important prognostic marker for mantle cell lymphoma and is associated with tumour aggressiveness [33]. Unfortunately, we did not validate this gene in drug-resistant cell lines, probably because of the influence of the in vitro culture environment on the transcriptome of cells or because the cell lines differ significantly from tumour primary cells in terms of genetic background.

The following are some of the study's limitations: First, all of the patient records included in the study were obtained from the GEO database; second, the lack of some crucial clinical data hindered further investigation. BM-MSCs from healthy individuals, cultured in vitro and proven to be senescent, were chosen as targets for differential gene selection in this work because their transcriptomes were influenced by tumour cells in vivo. Only one study's selection of the MSC gene for ageing may have resulted in biased gene selection, while several studies may have introduced batch-to-batch variation.

This is the first study to use the intersection of genes in ageing MSCs with differential genes in myeloma to find biomarkers that represent prognosis. We found genes in tumour cells with the same expression trend observed in MSC senescence that may provide targets for developing therapies capable of treating tumours while improving the ageing status of MSCs. Our research may help advance the development of drugs that not only kill MM cells but also improve the microenvironment. We offer new insights into the clinical diagnosis and treatment of MM, as well as suggestions for further research into the pathogenic mechanisms of drug resistance in myeloma. Overall, this research marks a step towards the ideal of data-driven clinical decision-making, in which better MM treatment options are determined using statistical models.

## 5. Conclusion

This research uncovered a collection of genes associated with ageing in MSCs that were linked to MM prognosis. The prognostic model was also constructed by incorporating clinical characteristics, which will facilitate individualized treatment. Moreover, the combination of drugs targeting lipid metabolism is expected to be a better treatment option for our predicted high-risk patients.

## Figures and Tables

**Table 1:** Primer sequences.

Genes	Primer
GAPDH	F: GGAAGCTTGTCATCAATGGAAATC
	R: TGATGACCCTTTTGGCTCCC

COBLL1	F: CAGATAAGAGTCCCTGTGAAGCA
	R: TGGCTGTAAGGCAGTCACACG

CCND1	F: AGCTGTGCATCTACACCGAC
	R: GAAATCGTGCGGGGTCATTG

MANSC1	F: CCTGTCCATTGAAACCAGCAA
	R: TCGGTGGGCTTTGAATAATCTG

MAN2A1	F: ACTATTTCGCCCTGAGACAAGC
	R: AAGTCTGGTACCATAATCCACAACC

EGR1	F: AAGGCCCTCAATACCAGCTAC
	R: ACTCCACTGGGCAAGCGTAA

HSPB8	F: AAGACCAAAGATGGATACGTGGAG
	R: AATGTTGAGTAAGGAGGGACCTG

SCN9A	F: ACAGCTTCTGCCAGAGGTGATA
	R: GAGGTTGGGATCATTCAGCATA

RGS7	F: TAAGATTCTGGCTGGCAGTGG
	R: CTCCTGAGCATCTTCAAATGTGTAT

NTF3	F: TTGCCACGATCTTACAGGTGAA
	R: TCCTTAACGTCCACCATCTGCT

OCLN	F: TTCCTATAAATCCACGCCGG
	R: TGTCTCAAAGTTACCACCGCTG

SLC31A2	F: GTGGTCATCGGCTACTTCATCAT
	R: CTGAGAAGTGGGTAAGCTAGGTAGTA

ZBED1	F: GAGGAGTGAGAATCAGAACCGC
	R: TGATGGTCTCCGCCGTGTT

SCN3A [1]	F: ATGTGGGACTGTATGGAGGTCG
	R: GGAAACACTCCCGCATCTTATT

SCN3A [2]	F: AAGAAATGCGGCAAGCTCAA
	R: CGTCGTCCTCATCCAGAAACA

SOX11 [1]	F: TTCAGTTTCAGAGGTCGGGC
	R: TTCTGTGGTGGTGCCGTTAC

SOX11 [2]	F: AAGCCCAAAATGGACCCCT
	R: ATTTCTTGCTGGAGCCCTTG

**Table 2:** The list of lasso regression coefficients.

Lasso genes	Coefficients
COBLL1	−0.177149459
CCND1	−0.029966789
MANSC1	−0.244343196
MAN2A1	−0.213850062
EGR1	−0.008368273
SLC31A2	−0.212850188
HSPB8	0.104505406
SCN3A	0.108917827
SCN9A	0.124850287
SOX11	0.504817715
RGS7	0.242200199
NTF3	0.194016607
OCLN	−0.341804389
ZBED1	0.236016978

## Data Availability

Raw data used to support the findings of this study are available at https://www.jianguoyun.com/p/DSmHWXMQzd_XChi70McEIAA.

## Supplementary Materials

Supplementary Materials The additional supplemental figure showed survival analysis of records from GEO accession numbers GSE83503 and GSE4581 grouped by the risk model. The validation set (GSE2658) Kaplan–Meier curve (A) and the validation set (GSE4581) Kaplan–Meier curve (B).
