# Supplementary material for: MSC Senescence-Related Genes Are Associated with Myeloma Prognosis and Lipid Metabolism-Mediated Resistance to Proteasome Inhibitors
Source: J Oncol. 2022 Nov 23;2022:4705654. doi: 10.1155/2022/4705654 (PMC9711959; doi:10.1155/2022/4705654)
Supplement: Supplementary Materials — The additional supplemental figure showed survival analysis of records from GEO accession numbers GSE83503 and GSE4581 grouped by the risk model. The validation set (GSE2658) Kaplan–Meier curve (A) and the validation set (GSE4581) Kaplan–Meier curve (B). [file 4705654.f1.pdf]

A

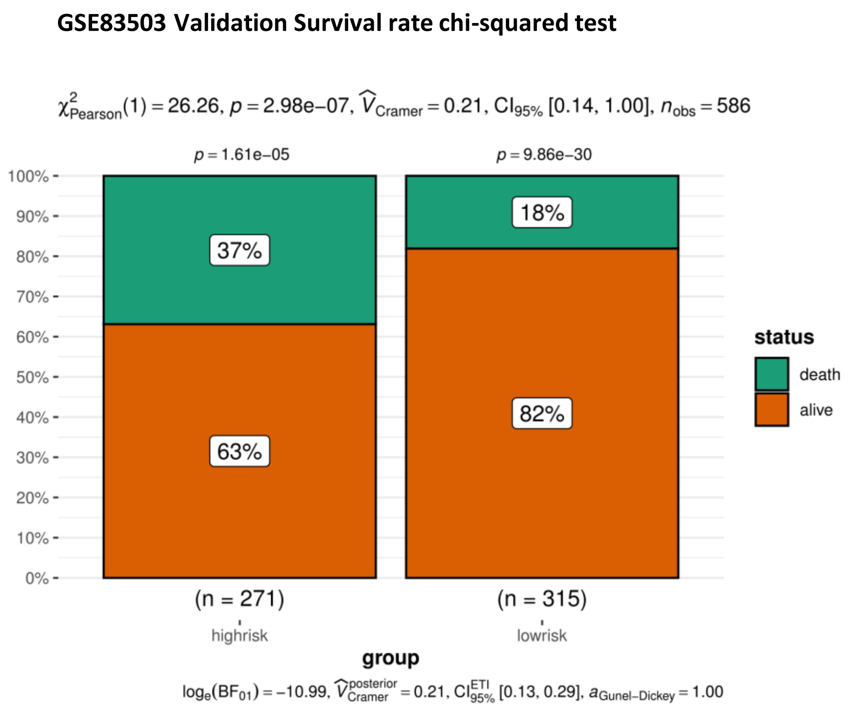

B

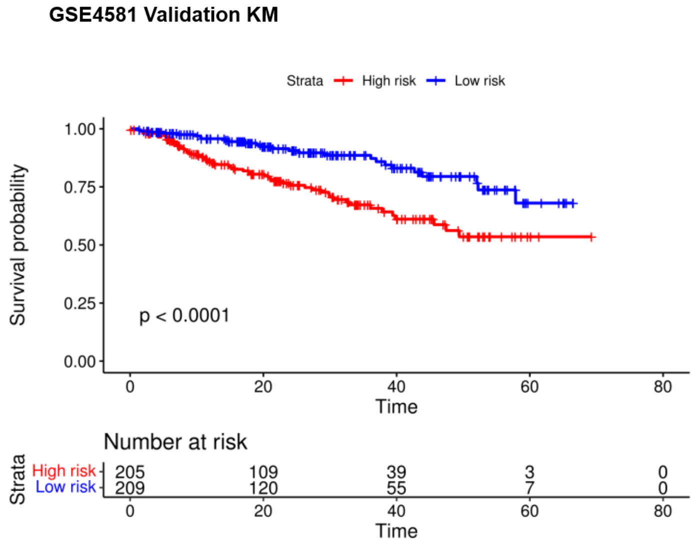

Figure S1. Survival analysis of records from GEO accession numbers GSE83503 and GSE4581 grouped by risk model. (A) A chi-square test was used to calculate the survival rate of the high- and low-risk groups in validation set (GSE83503), and the results showed that the mortality rate was significantly higher in the high-risk group than in the low-risk group ($P < 0.05$). (B) The validation set (GSE4581) Kaplan–Meier curve.
